# Crystal structure of a new mixed-metal coordin­ation polymer consisting of Ni^II^ piperidine-di­thio­carbamate and penta­nuclear Cu^I^—I cluster units

**DOI:** 10.1107/S2056989018000750

**Published:** 2018-01-26

**Authors:** Kento Himoto, Toshiya Horii, Shoki Oda, Shimpei Suzuki, Kunihisa Sugimoto, Takashi Okubo, Masahiko Maekawa, Takayoshi Kuroda-Sowa

**Affiliations:** a Department of Chemistry, Kindai University, Higashi-Osaka, Osaka 577-8502, Japan; b Research and Utilization Division, Japan Synchrotron Radiation Research Institute, Kouto, Sayo-cho, Sayo-gun, Hyogo 679-5198, Japan; c Research Institute for Science and Technology, Kindai University, Higashi-Osaka, Osaka 577-8502, Japan

**Keywords:** crystal structure, nickel(II), copper(I), heterometal, coordination polymer, di­thio­carbamate

## Abstract

A new heterometallic Cu^I^–Ni^II^ coordination polymer, poly[[tetra-μ_3_-iodido-μ_2_-iodido-bis­(μ_3_-piperidine-1-di­thio­carbamato)propio­nitrilepenta­copper(I)nickel(II)] chloro­form monosolvate], {[Cu^I^_5_Ni^II^I_5_(C_6_H_10_NS_2_)_2_(C_3_H_5_N)]·CHCl_3_}_*n*_, has been synthesized and structurally characterized. This coordination polymer consists of an Ni^II^ mononuclear unit of Ni^II^(Pip-dtc)_2_ (Pip-dtc^−^ is piperidine-1-di­thio­carbamate) and a penta­nuclear copper(I) cluster unit of Cu_5_I_5_(CH_3_CH_2_CN). The Ni^II^ ion, which lies on an inversion centre, is surrounded by four S atoms in a square-planar coordination geometry while all Cu^I^ ions have distorted tetra­hedral coordination geometries. In the penta­nuclear copper(I) cluster unit, a mirror plane passes through one Cu^I^ ion and three I ions. All the S atoms in Ni^II^(Pip-dtc) are also coordinated by the Cu^I^ ions, forming an infinite zigzag chain structure along the *b-*axis direction. The chains are weakly connected by solvent CHCl_3_ mol­ecules *via* Cl⋯I [3.653 (1) Å] and Cl⋯S [3.4370 (1) Å] short-contact inter­actions.

## Abstract 2

A novel heterometallic Cu^I^–Ni^II^ coordination polymer with an infinite one-dimensional structure was prepared and structurally characterized *via* X-ray diffraction. This complex consists of a mononuclear nickel(II) unit of Ni^II^(C_6_H_10_NS_2_)_2_ and a penta­nuclear copper(I) cluster unit of Cu_5_I_5_(CH_3_CH_2_CN).

## Chemical context   

The crystal engineering of coordination polymers is one of the most attractive areas in the field of materials science because their characteristic assembled structures and electronic states bearing features of organic–inorganic hybrid materials have new chemical and/or physical properties such as catalytic activity (Yaghi *et al.*, 2003 ▸), gas adsorption (Kitagawa *et al.*, 2004 ▸), conductivity (Givaja *et al.*, 2012 ▸), magnetism (Sato *et al.*, 1996 ▸) and optical properties (Watanabe *et al.*, 2017 ▸). The design and synthesis of coordination polymers have drawn much inter­est; in particular, the establishment of a rational synthetic method for preparing heterometallic coordination polymers is important in developing the chemistry of coordination complexes because of the unique coordination networks created by the combination of several metal ions with versatile coordination geometries (Ghosh *et al.*, 2018 ▸). Metal complexes with di­thio­carbamate (dtc) derivatives are some of the most useful building units to form heterometallic coordination polymers (Engelhardt *et al.*, 1988 ▸, 1989 ▸; Healy *et al.*, 1989 ▸; Tokoro *et al.*, 1995 ▸; Okubo *et al.*, 2012 ▸) because one can employ a variety of mononuclear metal complexes as building units for coordination polymers owing to the coord­ination ability of the sulfur atoms in the di­thio­carbamate complexes. In this paper, we report the synthesis and X-ray crystal structure of the title new heterometallic Cu^I^–Ni^II^ coordination polymer.
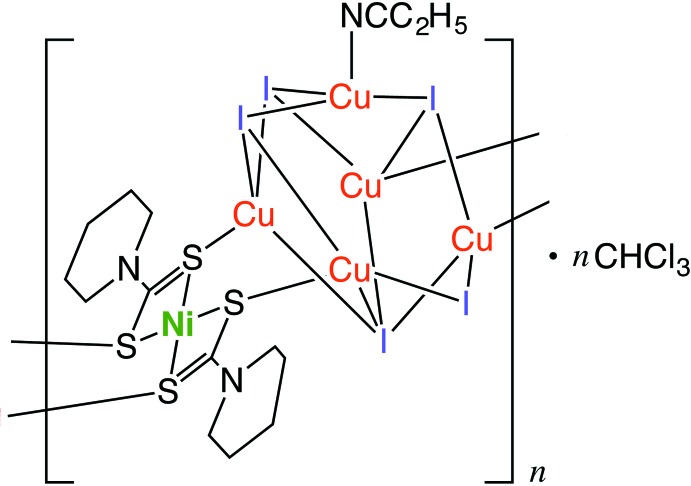


## Structural commentary   

**Figure 1:**
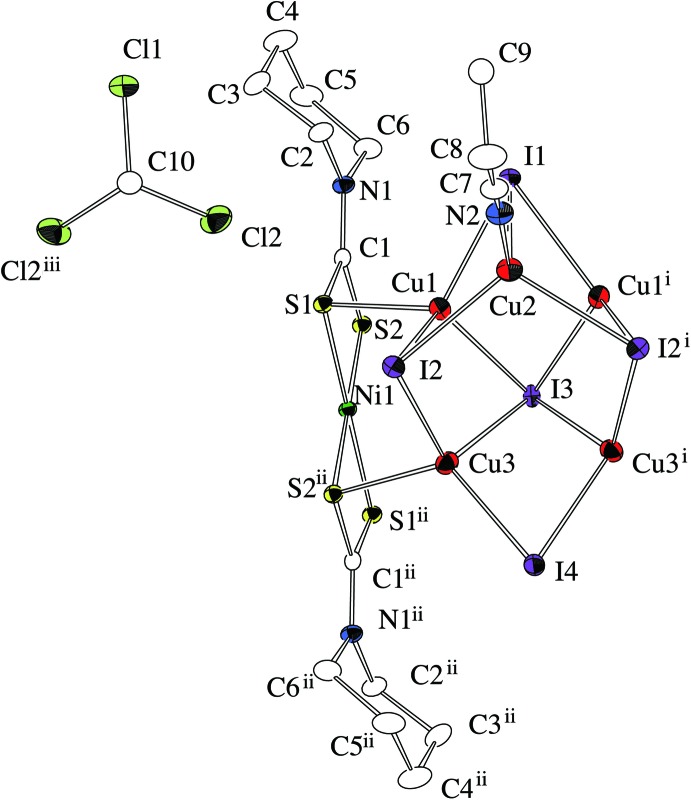
An *ORTEP* view of the title compound, showing the mononuclear Ni^II^ di­thio­carbamate unit Ni^II^(Pip-dtc)_2_, the penta­nuclear Cu^I^ cluster unit Cu_5_I_5_(CH_3_CH_2_CN) and the chloro­form mol­ecule with 50% probability level ellipsoids. H atoms have been omitted for clarity. [Symmetry codes: (i) *x*, −*y* + 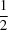, *z*; (ii) −*x*, −*y* + 1, −*z*; (iii) *x*, −*y* + 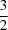, *z*.]

**Figure 2:**
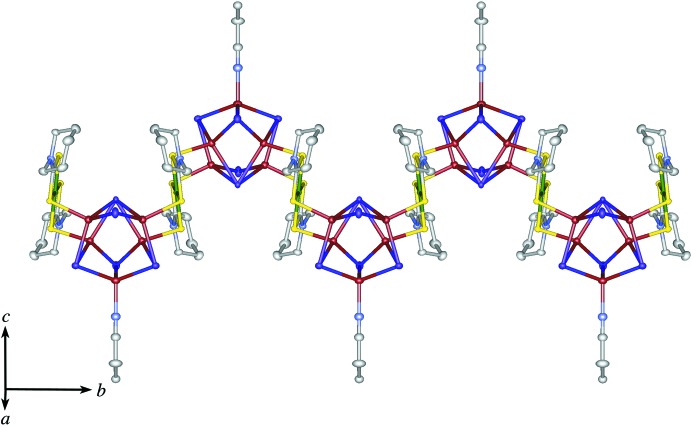
A packing diagram of the title compound, showing the one-dimensional chain structure: Cu red–brown, Ni green, I purple, S yellow, C white, N blue. H atoms and CH_3_Cl mol­ecules have been omitted for clarity.

The title compound (Fig. 1 ▸) has an infinite chain structure consisting of a mononuclear Ni^II^ di­thio­carbamate unit Ni^II^(Pip-dtc)_2_ (Pip–dtc^−^ = piperidine-di­thio­carbamate) and a penta­nuclear Cu^I^ cluster unit Cu_5_I_5_(CH_3_CH_2_CN). The Ni^II^ ion, which lies on an inversion centre, is surrounded by four S atoms from the dithiocarbamate ligands in a square-planar coordination geometry. The four S atoms in Ni^II^(Pip-dtc)_2_ are also coordinated by the Cu^I^ ions in the Cu^I^ cluster unit, forming an infinite zigzag chain along the *b-*axis direction (Fig. 2 ▸). In the Cu^I^ cluster unit, a mirror plane passes through one Cu^I^ ion (Cu2) and three I ions (I1, I3 and I4). The five Cu^I^ ions in the cluster create a distorted square-pyramidal structure bridged by five iodide ions, where four Cu^I^ ions [Cu1, Cu1^i^, Cu3 and Cu3^i^; symmetry code: (i) *x*, −*y* + 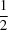, *z*] construct the basal plane and atom Cu2 is in the apical position. Atom I3 bridges the four basal Cu^I^ ions to stabilize the plane structure, while atoms I1 and I2 each bridge the two basal Cu^I^ ions and the apical Cu^I^ ion (Cu2). Atom I4 bridges the two basal Cu^I^ ions (Cu3 and Cu3^i^). One propionitrile ligand is coordinated to the apical Cu^I^ ion. In this cluster, the Cu1⋯Cu2 and Cu1⋯Cu3 distances of 2.6920 (6) and 2.7883 (3) Å, respectively, are shorter than the sum of the van der Waals radii for Cu⋯Cu (2.80 Å). In order to confirm the oxidation state of the copper ions, a bond-valence-sum (BVS) calculation was performed (Brese & O’Keeffe, 1991 ▸). The estimated BVS values for atoms Cu1, Cu2 and Cu3 are 1.08, 1.10 and 1.08, respectively, indicating their monovalent oxidation states.

## Supra­molecular features   

**Figure 3:**
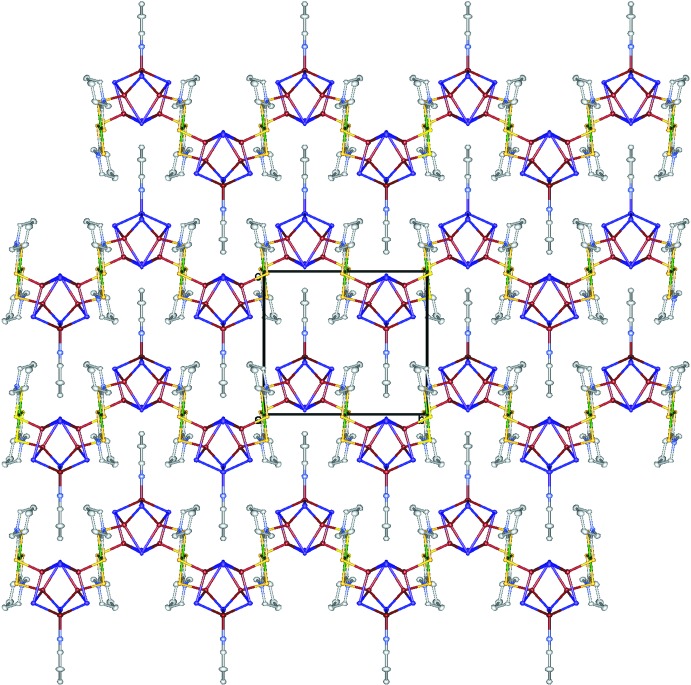
A packing diagram of the title compound viewed along the *a* axis: Cu red–brown, Ni green, I purple, S yellow, C white, N blue. H atoms and CH_3_Cl mol­ecules have been omitted for clarity.

**Figure 4:**
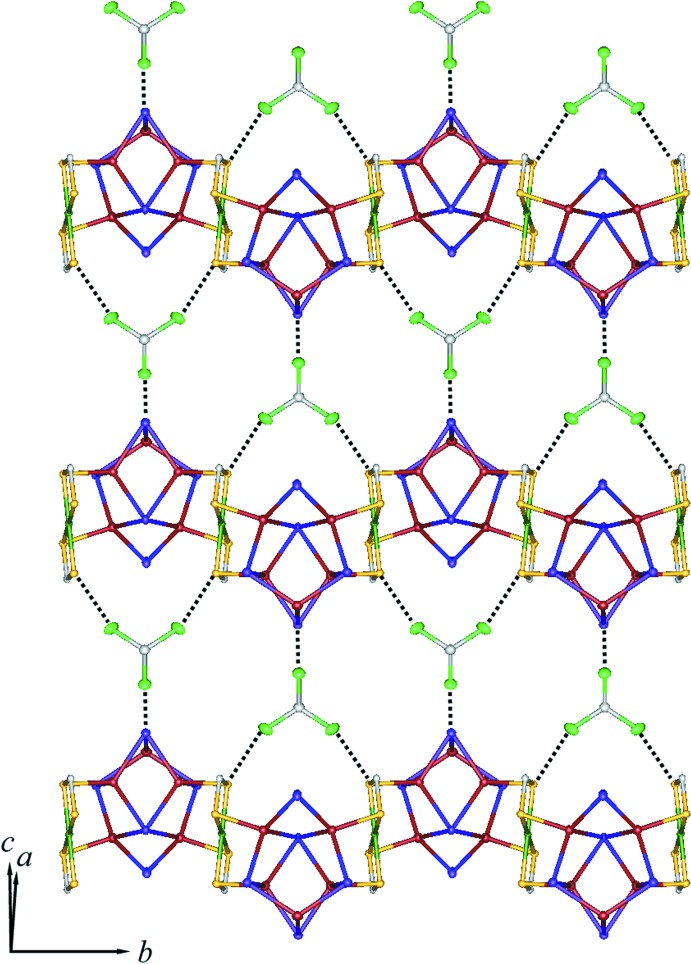
A packing diagram of the title compound, showing chains connected by weak Cl⋯S and Cl⋯I inter­actions (dashed lines). Piperidine groups of Pip-dtc ligands and H atoms have omitted for clarity.

Fig. 3 ▸ shows a packing diagram of zigzag chains alternately injected. The shortest I⋯I and I⋯S separations between the chains are 4.8100 (3) and 6.6517 (3) Å, respectively, which are greater than the sums of the van der Waals radii for I⋯I (3.96 Å) and I⋯S (3.78 Å). These chains are connected by solvent CHCl_3_ mol­ecules *via* Cl⋯I [3.653 (1) Å] and Cl⋯S [3.4370 (1) Å] contacts (Fig. 4 ▸), which are shorter than the sums of the van der Waals radii for Cl⋯I (3.73 Å) and Cl⋯S (3.55 Å), forming an undulating sheet parallel to (10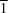).

## Spectroscopic properties   

**Figure 5:**
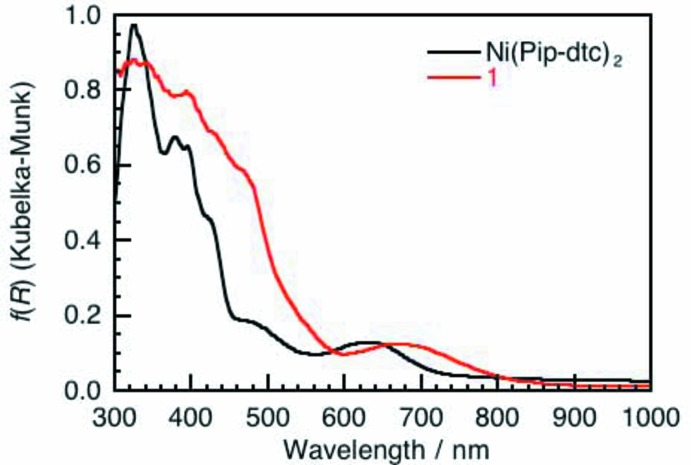
Diffuse–reflection UV–vis–NIR absorption spectra of mononuclear complex Ni(Pip–dtc)_2_ and coordination polymer **1** (0.01 mmol) doped in MgO powder (80 mg) obtained *via* the Kubelka–Munk analysis of reflectance spectra.

UV–vis–NIR spectra of the mononuclear Ni^II^ di­thio­carbamate complex, Ni^II^(Pip-dtc)_2_, and the title coordination polymer, **1**, were acquired using a U–4100 UV/VIS/NIR Spectrophotometer (HITACHI). Fig. 5 ▸ shows the diffuse-reflection spectra converted from the diffusion-reflectance (*R*) spectra using the Kubelka–Munk function: *f*(*R*) = (1 − *R*)^2^/2*R* (Kubelka, 1948 ▸). Ni^II^(Pip-dtc)_2_ shows two small absorption bands originating from the *d*–*d* transition of the Ni^II^ ion at 480 and 630 nm, as well as large absorption bands based on the charge-transfer transitions in the region of wavelengths less than 450 nm. On the other hand, **1** shows an absorption band at 680 nm, close to the wavelength (630 nm) of the *d*–*d* trans­ition of Ni^II^(Pip-dtc)_2_, but the absorption edge of the *d*–*d* transition shifts to the NIR region because of the formation of the energy band structure.

## Database survey   

A search of the Cambridge Structural Database (version 5.38, update May 2017; Groom *et al.*, 2016 ▸) for heterometallic coordination polymers with transition metal dithiocarbamate complexes and bridging copper-halides gave 11 hits: four heterometallic Co–Cu coordination polymers [refcodes GIJDEI and GIJDIM (Engelhardt *et al.*, 1988 ▸), SATWOZ and SATWUF (Healy *et al.*, 1989 ▸)], two heterometallic Cr–Cu coordination polymers (refcodes KEBREO and KEBRIS; Engelhardt *et al.*, 1989 ▸), two heterometallic Ni–Cu coordination polymers (refcodes UZENIY and UZENOE; Okubo *et al.*, 2012 ▸), two heterometallic Pt–Cu coordination polymers (refcodes ZENDAX and ZENDEB; Tokoro *et al.*, 1995 ▸), and one heterometallic Rh–Cu coordination polymer (refcode KEBRAK; Engelhardt *et al.*, 1989 ▸).

## Synthesis and crystallization   

The title compound was synthesized by the reaction of a CHCl_3_ solution (20 mL) of Ni^II^(Pip-dtc)_2_ (0.114 g, 0.1 mmol) and a 1:1 acetone/propio­nitrile solution (20 mL) of CuI (0.042g, 0.6 mmol). The reaction mixture was filtered, and dark-orange **[black in CIF?]** single crystals were obtained after letting the filtered solution stand for one day at room temperature. Yield: 56.7%. Analysis calculated for C_16_H_26_Cl_3_Cu_5_I_5_N_3_NiS_4_: C 12.76, H 1.74, N 2.79%; found: C 12.86, H 2.02, N 2.76%.

## Refinement details   

Crystal data, data collection and structure refinement details are summarized in Table 1 ▸. H atoms were located in a difference-Fourier map and then they were treated as constrained or restrained atoms.

## Supplementary Material

Crystal structure: contains datablock(s) global, I. DOI: 10.1107/S2056989018000750/is5484sup1.cif

Structure factors: contains datablock(s) I. DOI: 10.1107/S2056989018000750/is5484Isup3.hkl

CCDC reference: 1816493

Additional supporting information:  crystallographic information; 3D view; checkCIF report

## Figures and Tables

**Table 1:** Experimental details

Crystal data	
Chemical formula	[Cu_5_NiI_5_(C_6_H_10_NS_2_)_2_(C_3_H_5_N)]·CHCl_3_
*M*_r_	1505.90
Crystal system, space group	Monoclinic, *P*2_1_/*m*
Temperature (K)	100
*a*, *b*, *c* (Å)	11.6906 (4), 13.2597 (3), 12.6351 (4)
β (°)	112.829 (4)
*V* (Å^3^)	1805.19 (11)
*Z*	2
Radiation type	Mo *K*α
μ (mm^−1^)	8.15
Crystal size (mm)	0.10 × 0.05 × 0.02

Data collection	
Diffractometer	Rigaku XtaLAB P200
Absorption correction	Multi-scan (*CrysAlis PRO*; Rigaku Oxford Diffraction, 2015 ▸)
*T*_min_, *T*_max_	0.547, 0.850
No. of measured, independent and observed [*I* > 2σ(*I*)] reflections	22696, 5667, 5007
*R*_int_	0.037
(sin θ/λ)_max_ (Å^−1^)	0.734

Refinement	
*R*[*F* ^2^ > 2σ(*F* ^2^)], *wR*(*F* ^2^), *S*	0.021, 0.048, 1.02
No. of reflections	5667
No. of parameters	192
No. of restraints	6
H-atom treatment	H atoms treated by a mixture of independent and constrained refinement
Δρ_max_, Δρ_min_ (e Å^−3^)	2.37, −1.09

Computer programs: *CrysAlis PRO* (Rigaku Oxford Diffraction, 2015 ▸), *SHELXT* (Sheldrick, 2015*a* ▸), *SHELXL2017* (Sheldrick, 2015*b* ▸) and *CrystalStructure* (Rigaku, 2017 ▸).

## supplementary crystallographic information

### Crystal data

**Table:**

[Cu_5_NiI_5_(C_6_H_10_NS_2_)_2_(C_3_H_5_N)]·CHCl_3_	*F*(000) = 1392
*M**_r_* = 1505.90	*D*_x_ = 2.770 Mg m^−^^3^
Monoclinic, *P*2_1_/*m*	Mo *K*α radiation, λ = 0.71073 Å
*a* = 11.6906 (4) Å	Cell parameters from 10013 reflections
*b* = 13.2597 (3) Å	θ = 3.5–31.7°
*c* = 12.6351 (4) Å	µ = 8.15 mm^−^^1^
β = 112.829 (4)°	*T* = 100 K
*V* = 1805.19 (11) Å^3^	Block, black
*Z* = 2	0.10 × 0.05 × 0.02 mm

### Data collection

**Table:**

Rigaku XtaLAB P200 diffractometer	5007 reflections with *I* > 2σ(*I*)
Detector resolution: 5.811 pixels mm^-1^	*R*_int_ = 0.037
ω scans	θ_max_ = 31.5°, θ_min_ = 3.0°
Absorption correction: multi-scan (*CrysAlis PRO*; Rigaku Oxford Diffraction, 2015)	*h* = −16→15
*T*_min_ = 0.547, *T*_max_ = 0.850	*k* = −19→17
22696 measured reflections	*l* = −18→17
5667 independent reflections	

### Refinement

**Table:**

Refinement on *F*^2^	6 restraints
Least-squares matrix: full	Hydrogen site location: mixed
*R*[*F*^2^ > 2σ(*F*^2^)] = 0.021	H atoms treated by a mixture of independent and constrained refinement
*wR*(*F*^2^) = 0.048	*w* = 1/[σ^2^(*F*_o_^2^) + (0.0175*P*)^2^ + 1.401*P*] where *P* = (*F*_o_^2^ + 2*F*_c_^2^)/3
*S* = 1.02	(Δ/σ)_max_ = 0.001
5667 reflections	Δρ_max_ = 2.37 e Å^−^^3^
192 parameters	Δρ_min_ = −1.09 e Å^−^^3^

### Special details

**Table:**

Geometry. All esds (except the esd in the dihedral angle between two l.s. planes) are estimated using the full covariance matrix. The cell esds are taken into account individually in the estimation of esds in distances, angles and torsion angles; correlations between esds in cell parameters are only used when they are defined by crystal symmetry. An approximate (isotropic) treatment of cell esds is used for estimating esds involving l.s. planes.

### Fractional atomic coordinates and isotropic or equivalent isotropic displacement parameters (Å^2^)

**Table:**

	*x*	*y*	*z*	*U*_iso_*/*U*_eq_	
Cu1	0.07870 (3)	0.35696 (2)	0.22826 (3)	0.01492 (6)	
Cu2	0.06118 (4)	0.250000	0.40244 (4)	0.01831 (9)	
Cu3	−0.17631 (3)	0.36078 (2)	0.09941 (3)	0.01699 (6)	
I1	0.27076 (2)	0.250000	0.36316 (2)	0.01584 (5)	
I2	−0.07921 (2)	0.41447 (2)	0.31791 (2)	0.01423 (4)	
I3	−0.01641 (2)	0.250000	0.03136 (2)	0.01220 (5)	
I4	−0.37218 (2)	0.250000	0.04357 (2)	0.01957 (5)	
C1	0.2475 (2)	0.49357 (15)	0.13153 (19)	0.0107 (4)	
C2	0.4150 (2)	0.50565 (19)	0.32142 (19)	0.0160 (4)	
H2A	0.452733	0.443559	0.364245	0.019*	
H2B	0.346889	0.526878	0.344631	0.019*	
C3	0.5122 (3)	0.5885 (2)	0.3494 (2)	0.0232 (5)	
H3A	0.547669	0.600974	0.433227	0.028*	
H3B	0.472716	0.651752	0.310579	0.028*	
C4	0.6153 (2)	0.5587 (2)	0.3103 (2)	0.0261 (6)	
H4A	0.675553	0.614794	0.325656	0.031*	
H4B	0.659849	0.499170	0.354421	0.031*	
C5	0.5627 (2)	0.5341 (2)	0.1827 (2)	0.0230 (5)	
H5A	0.630165	0.508520	0.160911	0.028*	
H5B	0.529464	0.596390	0.138249	0.028*	
C6	0.4598 (2)	0.4552 (2)	0.1520 (2)	0.0180 (5)	
H6A	0.420231	0.447714	0.067571	0.022*	
H6B	0.495615	0.389276	0.185189	0.022*	
C7	0.1007 (3)	0.250000	0.6602 (3)	0.0204 (7)	
C8	0.1078 (4)	0.250000	0.7786 (3)	0.0293 (9)	
H8	0.0609 (16)	0.18967 (4)	0.785 (3)	0.035*	
C9	0.2415 (3)	0.250000	0.8654 (3)	0.0208 (7)	
H9A	0.239 (2)	0.250000	0.9419 (10)	0.031*	
H9B	0.2854 (9)	0.31033 (4)	0.8565 (13)	0.031*	
C10	0.2395 (3)	0.750000	0.5209 (3)	0.0186 (7)	
H10	0.199610	0.750000	0.578017	0.022*	
N1	0.36544 (17)	0.48550 (15)	0.19678 (16)	0.0126 (4)	
N2	0.0960 (3)	0.250000	0.5678 (3)	0.0208 (6)	
S1	0.13029 (5)	0.51728 (4)	0.18070 (5)	0.01093 (10)	
S2	−0.18105 (5)	0.52020 (4)	0.01647 (5)	0.01091 (10)	
Cl1	0.40231 (9)	0.750000	0.59606 (10)	0.0355 (2)	
Cl2	0.19291 (7)	0.64075 (6)	0.43626 (7)	0.03187 (16)	
Ni1	0.000000	0.500000	0.000000	0.00975 (8)	

### Atomic displacement parameters (Å^2^)

**Table:**

	*U*^11^	*U*^22^	*U*^33^	*U*^12^	*U*^13^	*U*^23^
Cu1	0.01456 (13)	0.01491 (13)	0.01762 (14)	0.00098 (11)	0.00879 (11)	0.00196 (11)
Cu2	0.0192 (2)	0.0224 (2)	0.01331 (18)	0.000	0.00625 (16)	0.000
Cu3	0.01664 (14)	0.01694 (13)	0.01690 (14)	−0.00328 (11)	0.00597 (11)	0.00057 (11)
I1	0.00947 (9)	0.01538 (9)	0.02001 (10)	0.000	0.00278 (7)	0.000
I2	0.01474 (7)	0.01642 (7)	0.01366 (7)	0.00173 (5)	0.00783 (5)	−0.00004 (5)
I3	0.01564 (10)	0.00914 (8)	0.01352 (9)	0.000	0.00751 (7)	0.000
I4	0.01104 (10)	0.01378 (9)	0.03178 (12)	0.000	0.00600 (9)	0.000
C1	0.0116 (10)	0.0087 (9)	0.0119 (9)	−0.0013 (7)	0.0046 (8)	0.0007 (7)
C2	0.0137 (10)	0.0211 (11)	0.0115 (10)	−0.0031 (9)	0.0030 (8)	−0.0021 (8)
C3	0.0184 (12)	0.0244 (13)	0.0228 (13)	−0.0074 (10)	0.0036 (10)	−0.0061 (10)
C4	0.0134 (11)	0.0374 (15)	0.0252 (13)	−0.0086 (11)	0.0049 (10)	0.0037 (12)
C5	0.0120 (11)	0.0350 (14)	0.0224 (12)	−0.0010 (10)	0.0073 (9)	0.0076 (11)
C6	0.0119 (10)	0.0259 (12)	0.0175 (11)	0.0024 (9)	0.0070 (8)	−0.0015 (9)
C7	0.0152 (15)	0.0261 (17)	0.0176 (16)	0.000	0.0038 (13)	0.000
C8	0.0218 (18)	0.050 (3)	0.0166 (17)	0.000	0.0082 (14)	0.000
C9	0.0230 (18)	0.0214 (16)	0.0178 (16)	0.000	0.0079 (14)	0.000
C10	0.0164 (16)	0.0209 (16)	0.0188 (16)	0.000	0.0070 (13)	0.000
N1	0.0090 (8)	0.0164 (9)	0.0125 (8)	−0.0003 (7)	0.0043 (7)	−0.0020 (7)
N2	0.0186 (14)	0.0245 (15)	0.0178 (14)	0.000	0.0054 (11)	0.000
S1	0.0094 (2)	0.0128 (2)	0.0110 (2)	−0.00043 (19)	0.00435 (18)	−0.00117 (18)
S2	0.0106 (2)	0.0120 (2)	0.0101 (2)	−0.00024 (19)	0.00394 (18)	−0.00100 (18)
Cl1	0.0190 (4)	0.0285 (5)	0.0436 (6)	0.000	−0.0048 (4)	0.000
Cl2	0.0251 (3)	0.0354 (4)	0.0314 (3)	0.0004 (3)	0.0068 (3)	−0.0173 (3)
Ni1	0.00797 (17)	0.01081 (17)	0.00993 (17)	−0.00041 (14)	0.00288 (14)	−0.00080 (14)

### Geometric parameters (Å, º)

**Table:**

Cu1—S1	2.3505 (6)	C4—H4A	0.9900
Cu1—I2	2.6259 (3)	C4—H4B	0.9900
Cu1—I1	2.6435 (4)	C5—C6	1.526 (4)
Cu1—I3	2.7001 (4)	C5—H5A	0.9900
Cu2—N2	1.968 (3)	C5—H5B	0.9900
Cu2—I1	2.6820 (5)	C6—N1	1.476 (3)
Cu2—I2	2.6889 (3)	C6—H6A	0.9900
Cu2—I2^i^	2.6889 (3)	C6—H6B	0.9900
Cu3—S2	2.3505 (6)	C7—N2	1.147 (5)
Cu3—I4	2.5778 (4)	C7—C8	1.466 (5)
Cu3—I2	2.6429 (4)	C8—C9	1.521 (5)
Cu3—I3	2.7637 (4)	C8—H8	0.9901 (10)
C1—N1	1.307 (3)	C8—H8^i^	0.9901 (10)
C1—S2^ii^	1.734 (2)	C9—H9A	0.9800 (10)
C1—S1	1.739 (2)	C9—H9B	0.9800 (10)
C2—N1	1.476 (3)	C9—H9B^i^	0.9800 (10)
C2—C3	1.521 (3)	C10—Cl2	1.757 (2)
C2—H2A	0.9900	C10—Cl2^iii^	1.757 (2)
C2—H2B	0.9900	C10—Cl1	1.767 (4)
C3—C4	1.523 (4)	C10—H10	1.0000
C3—H3A	0.9900	S1—Ni1	2.2106 (5)
C3—H3B	0.9900	S2—Ni1	2.2219 (5)
C4—C5	1.521 (4)		
			
Cu1···Cu2	2.6920 (5)	Cu1···Cu1^i^	2.8366 (6)
Cu1···Cu3	2.7883 (4)	Cu3···Cu3^i^	2.9378 (6)
			
S1—Cu1—I2	98.089 (17)	S2^ii^—C1—S1	108.58 (12)
S1—Cu1—I1	114.335 (17)	N1—C2—C3	109.1 (2)
I2—Cu1—I1	116.170 (13)	N1—C2—H2A	109.9
S1—Cu1—Cu2	142.553 (19)	C3—C2—H2A	109.9
I2—Cu1—Cu2	60.730 (11)	N1—C2—H2B	109.9
I1—Cu1—Cu2	60.344 (12)	C3—C2—H2B	109.9
S1—Cu1—I3	107.115 (17)	H2A—C2—H2B	108.3
I2—Cu1—I3	116.286 (12)	C2—C3—C4	110.6 (2)
I1—Cu1—I3	104.798 (11)	C2—C3—H3A	109.5
Cu2—Cu1—I3	109.985 (12)	C4—C3—H3A	109.5
S1—Cu1—Cu3	99.008 (17)	C2—C3—H3B	109.5
I2—Cu1—Cu3	58.346 (10)	C4—C3—H3B	109.5
I1—Cu1—Cu3	146.557 (14)	H3A—C3—H3B	108.1
Cu2—Cu1—Cu3	94.797 (15)	C5—C4—C3	110.8 (2)
I3—Cu1—Cu3	60.446 (10)	C5—C4—H4A	109.5
S1—Cu1—Cu1^i^	154.742 (15)	C3—C4—H4A	109.5
I2—Cu1—Cu1^i^	106.881 (7)	C5—C4—H4B	109.5
I1—Cu1—Cu1^i^	57.554 (7)	C3—C4—H4B	109.5
Cu2—Cu1—Cu1^i^	58.208 (8)	H4A—C4—H4B	108.1
I3—Cu1—Cu1^i^	58.314 (7)	C4—C5—C6	111.8 (2)
Cu3—Cu1—Cu1^i^	91.040 (8)	C4—C5—H5A	109.3
N2—Cu2—I1	111.67 (9)	C6—C5—H5A	109.3
N2—Cu2—I2	105.34 (5)	C4—C5—H5B	109.3
I1—Cu2—I2	112.776 (11)	C6—C5—H5B	109.3
N2—Cu2—I2^i^	105.34 (5)	H5A—C5—H5B	107.9
I1—Cu2—I2^i^	112.776 (12)	N1—C6—C5	110.5 (2)
I2—Cu2—I2^i^	108.395 (17)	N1—C6—H6A	109.6
N2—Cu2—Cu1	144.91 (4)	C5—C6—H6A	109.6
I1—Cu2—Cu1	58.933 (12)	N1—C6—H6B	109.6
I2—Cu2—Cu1	58.418 (9)	C5—C6—H6B	109.6
I2^i^—Cu2—Cu1	109.322 (16)	H6A—C6—H6B	108.1
N2—Cu2—Cu1^i^	144.90 (4)	N2—C7—C8	179.5 (4)
I1—Cu2—Cu1^i^	58.932 (12)	C7—C8—C9	111.8 (3)
I2—Cu2—Cu1^i^	109.322 (16)	C7—C8—H8	105.5 (19)
I2^i^—Cu2—Cu1^i^	58.419 (9)	C9—C8—H8	112.9 (16)
Cu1—Cu2—Cu1^i^	63.585 (16)	C7—C8—H8^i^	105.5 (19)
S2—Cu3—I4	121.711 (19)	C9—C8—H8^i^	112.9 (17)
S2—Cu3—I2	98.612 (17)	H8—C8—H8^i^	107.80 (19)
I4—Cu3—I2	114.539 (14)	C8—C9—H9A	107.0 (15)
S2—Cu3—I3	103.950 (17)	C8—C9—H9B	110.8 (8)
I4—Cu3—I3	104.370 (12)	H9A—C9—H9B	109.44 (16)
I2—Cu3—I3	113.573 (12)	C8—C9—H9B^i^	110.8 (8)
S2—Cu3—Cu1	96.405 (18)	H9A—C9—H9B^i^	109.44 (16)
I4—Cu3—Cu1	141.671 (14)	H9B—C9—H9B^i^	109.4 (2)
I2—Cu3—Cu1	57.751 (10)	Cl2—C10—Cl2^iii^	111.1 (2)
I3—Cu3—Cu1	58.198 (10)	Cl2—C10—Cl1	110.00 (14)
S2—Cu3—Cu3^i^	154.073 (15)	Cl2^iii^—C10—Cl1	110.00 (14)
I4—Cu3—Cu3^i^	55.263 (8)	Cl2—C10—H10	108.6
I2—Cu3—Cu3^i^	105.626 (7)	Cl2^iii^—C10—H10	108.6
I3—Cu3—Cu3^i^	57.894 (7)	Cl1—C10—H10	108.6
Cu1—Cu3—Cu3^i^	88.961 (8)	C1—N1—C2	122.64 (19)
Cu1—I1—Cu1^i^	64.893 (14)	C1—N1—C6	122.8 (2)
Cu1—I1—Cu2	60.724 (11)	C2—N1—C6	114.55 (18)
Cu1^i^—I1—Cu2	60.724 (11)	C7—N2—Cu2	171.6 (3)
Cu1—I2—Cu3	63.902 (10)	C1—S1—Ni1	86.27 (7)
Cu1—I2—Cu2	60.851 (12)	C1—S1—Cu1	104.11 (7)
Cu3—I2—Cu2	98.341 (12)	Ni1—S1—Cu1	91.60 (2)
Cu1—I3—Cu1^i^	63.372 (13)	C1^ii^—S2—Ni1	86.04 (8)
Cu1—I3—Cu3	61.356 (10)	C1^ii^—S2—Cu3	108.01 (7)
Cu1^i^—I3—Cu3	94.530 (11)	Ni1—S2—Cu3	94.29 (2)
Cu1—I3—Cu3^i^	94.530 (11)	S1—Ni1—S1^ii^	180.0
Cu1^i^—I3—Cu3^i^	61.357 (10)	S1—Ni1—S2^ii^	79.02 (2)
Cu3—I3—Cu3^i^	64.211 (13)	S1^ii^—Ni1—S2^ii^	100.98 (2)
Cu3—I4—Cu3^i^	69.475 (15)	S1—Ni1—S2	100.98 (2)
N1—C1—S2^ii^	126.40 (18)	S1^ii^—Ni1—S2	79.02 (2)
N1—C1—S1	125.01 (17)	S2^ii^—Ni1—S2	180.0
			
N1—C2—C3—C4	−57.4 (3)	C3—C2—N1—C1	−121.9 (2)
C2—C3—C4—C5	56.4 (3)	C3—C2—N1—C6	58.3 (3)
C3—C4—C5—C6	−53.4 (3)	C5—C6—N1—C1	124.8 (2)
C4—C5—C6—N1	51.7 (3)	C5—C6—N1—C2	−55.4 (3)
S2^ii^—C1—N1—C2	175.50 (17)	N1—C1—S1—Ni1	−176.18 (19)
S1—C1—N1—C2	−6.1 (3)	S2^ii^—C1—S1—Ni1	2.44 (9)
S2^ii^—C1—N1—C6	−4.7 (3)	N1—C1—S1—Cu1	−85.46 (19)
S1—C1—N1—C6	173.69 (18)	S2^ii^—C1—S1—Cu1	93.16 (10)

Symmetry codes: (i) *x*, −*y*+1/2, *z*; (ii) −*x*, −*y*+1, −*z*; (iii) *x*, −*y*+3/2, *z*.
